# A scoping review comparing individual and multi-level physical activity interventions in rural women in the United States

**DOI:** 10.1186/s12889-025-24018-y

**Published:** 2025-12-03

**Authors:** Katrina L. Wilhite, Alexis Thrower, Sarah Modlin, Meiling Zheng, Carly Williamson Rogers, Elly Marshall, Virginia Desouky, Christiaan G. Abildso, Cynthia K. Perry, M Renée Umstattd Meyer, Bethany Barone Gibbs

**Affiliations:** 1 Epidemiology and Biostatistics, West Virginia University, 64 Medical Center Drive, PO Box 9190, Morgantown, WV 26506 USA; 2 Exercise Physiology, West Virginia University, Morgantown, WV USA; 3 Exercise Physiology, University of Pittsburgh, Pittsburgh, PA USA; 4 Social and Behavioral Sciences, West Virginia University, Morgantown, WV USA; 5 West Virginia University Libraries, Morgantown, WV USA; 6 West Virginia University Extension, Morgantown, WV USA; 7 School of Nursing Oregon Health &, Science University Portland OR, Portland, USA; 8 Public Health, Baylor University, Waco, TX USA

**Keywords:** Physical activity, Women, Rural

## Abstract

**Background:**

Women residing in rural areas of the United States tend to have worse cardiovascular, mental, and pregnancy-related health than their urban counterparts. Physical activity is a modifiable and likely promising strategy to reduce these health disparities. The aim of this review is to summarize physical activity interventions in rural women in the US using the socio-ecological model of health framework.

**Methods:**

We conducted a scoping review of peer-reviewed literature in Medline, CINAHL Complete, Web of Science, and PsychINFO in August 2023. Studies had to be published in a peer-reviewed journal, quantitatively assess physical activity effects of an intervention in the US, include women who were on average aged 18 years or older and live in a rural setting, and be written in English. Studies that did not separately analyze the effects of physical activity interventions on rural, women were excluded. We narratively synthesized the evidence and, in studies with a comparison group, calculated pooled estimates to compare effects between individual vs multi-level approaches. Multi-level interventions were defined as those intervening at two or more levels of the socio-ecological model. We used an adapted GRADE approach to assess study quality.

**Results:**

Our search yielded 41 reports on 38 different interventions that met our eligibility criteria (24 individual and 17 multi-level). After initial completion of the intervention (n = 38), 19/23 individual (83%) and 11/15 multi-level (73%) interventions increased physical activity. Pooled estimates indicated that multi-level interventions increased physical activity to a greater extent (*d* 1.09 [-0.44, 2.16]) than individual-level interventions (*d* 0.18 [-0.26, 0.62]). Limited studies included measurements of feasibility, acceptability, strengths, and barriers or defined rurality, identifying a need to better report these measures. Considerations for designing interventions include ensuring that participants feel supported, employing strong interventionists, new technology, and cost-effectiveness.

**Conclusions:**

Our results found that more individual-level interventions increased physical activity compared to multi-level interventions. Yet, meta-analysis results suggest that multi-level interventions yielded a larger pooled effect than individual-level interventions; however, with overlapping confidence intervals, we cannot definitively claim that either approach was more successful. More high-quality interventions, including randomized controlled trials, are needed. Reporting should be detailed and thorough.

**Supplementary Information:**

The online version contains supplementary material available at 10.1186/s12889-025-24018-y.

## Background

Globally, reducing health disparities has been identified as a key health priority. The United Nations' fifth Sustainable Development Goal is to “achieve gender equality and empower all women and girls,” and the tenth goal is to “reduce inequality within and among countries” [1]. Specifically in the United States, the American Heart Association issued a call to action for rural populations in 2020, [2] followed by specific requests for research of women released by the National Institutes of Health in 2024 [3]. Although the cardiovascular mortality rate has progressively decreased in the general US population, it has increased among rural populations since 2009 [4]. Additionally, women residing in rural areas in the United States tend to experience worse mental and pregnancy-related health outcomes than their urban counterparts [5–10]. Cardiovascular disease is the leading cause of death in US women [11]. Furthermore, despite mortality rates decreasing over time, there has been a minimal decrease in this risk among women of reproductive age [12, 13]. Therefore, strategies to reduce health inequities to improve health, particularly cardiovascular health, in rural women need to be prioritized.

Participating in sufficient physical activity is currently defined by US guidelines as at least 150 min of moderate-intensity aerobic physical activity, 75 min of vigorous-intensity aerobic physical activity, or an equivalent combination of both per week, and strength training involving all major muscle groups at least two times per week [14]. Meeting these guidelines has many health benefits such as reduced risk for cardiovascular disease and better mental health [15–17]. Physical activity is an effective strategy to improve health, and it may be an attractive intervention option in rural communities because it is a modifiable and cost-effective behavioral target [18, 19]. Yet, analysis of National Health Interview Survey data shows that US adults in rural areas were 11–32% less likely to meet physical activity guidelines than those living in urban areas [20]. Additionally, across all age groups, US women are less likely to meet physical activity guidelines compared to men [21]. This may be due to specific barriers women face, including fears of personal safety when exercising alone, overcoming weight and body image stigmas, embarrassment, and social and internal pressures of prioritizing family over personal wellness [22]. This highlights the need for targeted female physical activity interventions in rural areas and an opportunity to reduce health disparities.

The factors that influence physical activity are complicated and occur at multiple levels [23]. The socio-ecological model of health integrates societal (e.g., structural, policy), organizational (e.g., worksite), community (e.g., church), interpersonal (e.g., family), and individual (e.g., motivation) levels to understand and promote healthy behaviors [24]. Further, it has been identified as a useful framework to effectively recruit and retain underserved communities in clinical trials [25]. Interventions in rural women that operate on multiple socio-ecological levels to increase physical activity may be an effective way to reduce health disparities in this priority population, as there are several physical activity barriers to address across levels including limited walkability and less social support [26, 27]. Effective interventions may include elements such as groups within the community to learn proper exercise techniques, coupled with health coaching sessions to identify strategies to increase family and peer support to lead a physically active lifestyle. Although previous reviews have focused on rural physical activity interventions, to our knowledge, none have exclusively focused on women [28, 29]. However, a comprehensive review of physical activity intervention designs and effectiveness in rural women does not exist. Therefore, this review aims to summarize the existing literature on interventions to increase physical activity in rural women in the US from a socio-ecological perspective.

## Methods

We prospectively registered this scoping review on Open Science Framework (https://osf.io/64q78/) and adhered to the Preferred Reporting Items for Systematic Reviews and Meta-Analyses extension for Scoping Reviews (PRISMA-ScR) [30] guidelines. Conducting a scoping review was appropriate to identify and summarize the interventions that are in the field; to examine how interventions are conducted in rural women in the US; to identify key strengths, barriers, feasibility, and acceptability of the interventions that have been implemented; and to identify gaps in the literature so that we can provide considerations for future intervention development [31].

To be included in this review, studies had to be published in a peer-reviewed journal, utilize quantitative data to assess physical activity effects of an intervention in the US, include women who were on average aged 18 years or older and live in a rural setting, and be written in English. All quantitative data collection methods (e.g., device-based or surveys), study designs, and publication years were considered eligible. There was no date restriction placed to provide a comprehensive summary of all available rural physical activity interventions in US women. Studies were excluded if they included both men and women participants, but the women’s data were not analyzed separately; included participants from rural, urban, and/or suburban populations, but were not analyzed separately by rurality; or presented results as a conference abstract, pre-print, or thesis/dissertation.

### Search strategy

To identify potentially relevant documents, electronic bibliographic databases were searched. The search strategies were drafted by an experienced medical librarian (VD). A separate librarian reviewed the search strategy using the PRESS checklist [32]. This search was applied to Medline (PubMed, 1966 – present), CINAHL Complete (1937—present), Web of Science (1900—present), and PsychINFO (1872 – present). The last search was run on August 22, 2023. The search string from PubMed used to identify studies of interest is reported in Additional File 1. The final search results were exported into EndNote.

### Article selection and data collection

All citations were uploaded and deduplicated in the Covidence reference management software (Covidence, Melbourne, Victoria, Australia (www.covidence.org)). Subsequently, all titles and abstracts were screened independently and in duplicate. Conflicts were immediately moved forward to the full-text review phase. Full texts were reviewed independently and in duplicate. If reviewers disagreed on the eligibility of a study, the conflicts were resolved by discussion until a consensus was reached. Additionally, to capture as many citations as possible, four studies found during preliminary searches were used for bi-directional screening, a method where one reviewer screens all references within an article and any articles that cited the article [33].

Data charting was completed with a customized data charting tool which was piloted by three reviewers and revised based on their feedback. Items included authors, year of publication, sample size, participant characteristics, eligibility criteria, rurality definition, region, study design, intervention details, delivery method (i.e., in-person, remote, hybrid), physical activity outcomes (e.g., moderate-to-vigorous intensity physical activity (MVPA), strength training), data collection methods (e.g., device-based measurement), intervention format (e.g., community-based, web platforms), at which socio-ecological level the intervention targeted (i.e., societal, organizational, community, interpersonal, individual, multi-level), reported feasibility, reported acceptability, reported strengths, reported barriers, and results. If the intervention was targeted at more than one level, such as improving exercise self-efficacy (individual) and providing strategies to enhance social support (interpersonal) the intervention would be considered multi-level. To be included in the synthesis, feasibility and acceptability had to include a reported measurement, such as the percentage of participants who found the intervention useful. Strengths and barriers were those reported by the authors of a specific intervention. All data charting was done independently and in duplicate. All discrepancies were reviewed and discussed until a consensus was reached.

Although not typical for a scoping review, we performed a critical appraisal of study quality at the study level using an adapted GRADE approach [34]. Studies are first ranked by their study design: randomized controlled trials are high quality, non-randomized or quasi-randomized controlled trials are moderate, and studies without a control group are low quality. Next, studies are upgraded, maintained, or downgraded based on factors such as allocation concealment, lack of blinding, loss to follow-up, and selective outcome reporting. We added factors relevant to our review aims, such as whether physical activity outcomes were collected subjectively or device-based and whether or not the intention-to-treat principle was followed. As this study aims to summarize interventions, reporting the study quality is essential to identifying critical gaps and appropriately reviewing and adapting the interventions that have been disseminated in the field. The critical appraisal was completed independently and in duplicate with conflicts resolved by discussion.

We have summarized the studies overall, including feasibility, acceptability, strengths, and barriers**,** and by each level of the socio-ecological model. Intervention effects were based on direction, not significance, as per Cochrane [35]. As interventions are detailed and clinically diverse, defined by Cochrane as “variability in participants, interventions and outcomes,” [35] we chose one intervention that increased physical activity as an exemplar study for each socio-ecological level. The exemplar study was chosen based on study quality, study design, and sample size.

### Quantitative analysis

We conducted two meta-analyses, one with individual-level and one with multi-level interventions, to better understand the effects at different levels. We considered performing a meta-analysis combining all interventions; however, due to the methodological diversity of interventions and the lower quality of studies included in the review, the total effect would not have been meaningful and potentially misleading. [36] Instead, we generated a forest plot with the results from each meta-analysis to compare the intervention effects at each level. Therefore, the results should only be used to compare potential differences in individual versus multi-level interventions rather than to estimate true intervention effects. Analyses were performed with MetaXL, version 5.3 (EpiGear International Pty Ltd., Brisbane, Australia; (https://www.epigear.com/index_files/metaxl.html) [37, 38] We only included studies with a comparison group to avoid biased results [39]. We used the quality effects model to pool results within each socio-ecological model. The quality effects model employs a random-effects model that also considers a quality score for weighting each study [40]. The benefit of including a quality score is that the model considers bias in studies such as attrition rate or data collection methods. For this study, quality scores were based on our quality assessment using an adapted GRADE approach. Quality scores per study were 0.25 (very low quality), 0.50 (low quality), 0.75 (moderate quality), and 1.00 (high quality).

Some studies reported on multiple physical activity outcomes. Therefore, we used a hierarchy of outcomes to select what would be entered into the model as follows: duration then volume of MVPA, duration then volume of moderate-intensity physical activity, and duration then volume of vigorous-intensity physical activity. We chose MVPA as our first ranking since this outcome was often reported, and the aerobic portion of the current US physical activity guidelines are based on this intensity of aerobic physical activity. We considered making a separate forest plot for strength training, but only two multi-level studies reported this outcome. In instances where a control group was being compared to multiple intervention variations, combined intervention means and standard deviations were calculated. When there were multiple studies reporting on the same intervention, the study that reported immediate post-intervention effects was used. In one case, an intervention started in pregnancy and continued postpartum, with effects reported during both time periods. We only included the study with the pregnant population since the reported effect used a larger sample.

## Results

### Overview

**Fig. 1:**
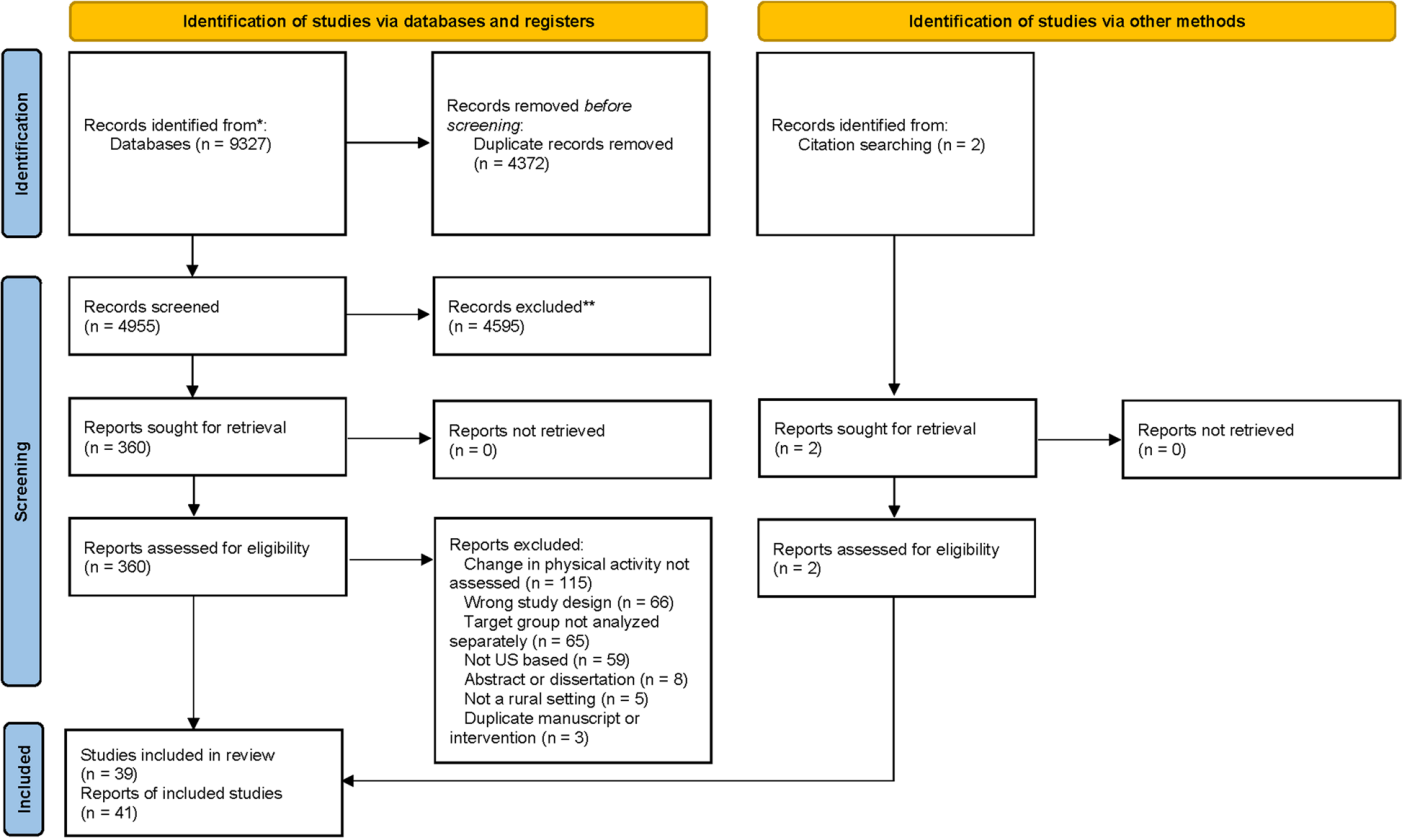
Article Selection

We included 41 studies that analyzed 38 different interventions that met our eligibility criteria (see Fig. 1 for details), all had interventions that were either individual or multi-level. No interventions were identified at the remaining levels of the socio-ecological model (e.g., interpersonal). Studies were published between 2002–2022 with interventions implemented between 2002–2017. Most studies were from New York (k = 7), North Carolina (k = 5), and the Midwest (k = 5). Rurality was defined by Rural–Urban Communing Area (RUCA) Code [41] (k = 8), Rural–Urban Continuum Codes [42] (RUCC, k = 4), population cut points (k = 4), a frontier county [43] (< = 6 people/square mile, k = 2), and as defined by the US Office of Management and Budget [44] (k = 1). The remaining 22 studies (53.7%) did not specify how they defined rurality. Physical activity outcomes included steps, light-intensity physical activity, moderate-intensity physical activity, vigorous-intensity physical activity, MVPA, strengthening exercises, stretching exercises, energy expenditure, meeting pre-defined physical activity recommendations, frequency of specific exercises (e.g., swimming, running, household-related), and general questions (e.g., “I exercised yesterday”). Physical activity was primarily measured via self-report (k = 26), followed by a combination of a device-based measurement and self-report (k = 7), pedometer (k = 6), and accelerometry (k = 3). Special populations included those with overweight/obesity (k = 10), underrepresented racial groups (k = 10), low socioeconomic status (k = 3), prenatal (k = 3), cancer survivors (k = 3), blue-collar workers (k = 2), elderly, prehypertensive, diagnosed with arthritis, and postpartum (k = 1 each). Specific study characteristics can be found in Table 1.

**Table 1:** Study Characteristics

Author (Year)	Design (Theory)	Population Description (Sample Size)	State/Region	Rurality Definition	Delivery Method (Location)	Intervention Name	Physical Activity Outcome(s)	Brief Results^a,b^
Individual (k = 24)								
Befort (2010) [45]	Pre-post (Social Cognitive Theory)	Overweight (34)	Kansas	Population < 10,000	Remote	None	METs	PA increased after 24 weeks
Befort (2012) [46]	Pre-post (Not reported)	Breast cancer survivors (35)	Kansas	RUCA	Remot	None	Energy expenditure & PA	Energy expenditure & PA increased after 6 months
Edwards (2021) [47]	Pre-post (Social Cognitive Theory, Self-Determination Theory)	Midlife (35)	Not reported	Not defined	In-person (Not reported)	Fit Minded	Steps	Steps increased after 12 weeks
Fazzino (2017) [48]	Pre-post (Not reported)	Postmenopausal breast cancer survivors (142)	Midwest	Not defined	Remote	None	MVPA, % participants meeting guidelines	MVPA increased at 6 months but was lower at 18 months compared to 6 months Increased % of participants meeting guidelines
Folta (2009) [49]	RCT (Not reported)	Sedentary, overweight and obese, midlife (96)	Arkansas and Kansas	Not defined	In-person (Not reported)	Strong Women—Healthy Hearts	Steps	Intervention Steps increased after 12 weeks Control Steps decreased after 12 weeks Intervention vs. control Intervention group reported more steps compared to the control group
Greaney (2017) [50]	RCT (Social Cognitive Theory)	Overweight or obese, African American (121)	North Carolina	Not defined	Hybrid (Not reported)	The Shape Program	MVPA, % of participants meeting MVPA guidelines	Intervention MVPA increased after 12 months but % of participants meeting guidelines decreased Control All measures of PA decreased after 12 months Intervention vs. control MVPA increased in the intervention vs. control group
Griffin (2018) [51]	Pre-post (Social Cognitive Theory)	Low-income (143)	Alabama	Not defined	Remote	My Quest	Response to “Yesterday, I exercised 30 min”	More participants reported exercising for 30 min the previous day
Hageman (2014) [52]	RCT (Pender’s Health Promotion Model)	Prehypertensive, midlife (289)	Nebraska	RUCA	Hybrid (Home)	Wellness for Women: DASHing towards Health	MVPA, energy expenditure, % participants meeting MVPA guidelines	Intervention All measures of PA increased after 12 months Control All measures of PA increased after 12 months Intervention vs. control No difference in PA change between groups
Hageman^c^ (2017) [53]	RCT (Pender’s Health Promotion Model)	Overweight or obese, aged 40–49 (301)	Nebraska	RUCA	Remote	Women Weigh-in for Wellness	MVPA, % participants meeting MVPA guidelines	All participants received some modified version of the intervention. There was no change in physical activity between groups after 6, 18, or 30 months
Hageman^c^ (2022) [54]	RCT (Pender’s Health Promotion Model)	Obesity and arthritis (82)	Nebraska	RUCA	Remote	Women Weigh-in for Wellness	MVPA	In a combined analysis, all participants increased their MVPA after 30 months compared to baseline
Khare (2021) [55]	Pre-post (Social Cognitive Theory)	Midlife (44)	Illinois	RUCC	Remote	Step-2-It	% participants meeting guidelines, steps	More people reported meeting MPA guidelines but steps decreased after 12 weeks
Maddock (2022) [56]	RCT (Not reported)	General^d^ (182)	New York	RUCA	In-person (Community spaces)	Strong Hearts, Healthy Communities, 2.0	LPA, MVPA, walking, % participants meeting guidelines	Intervention All PA measures increased after 6 months Control Mixed results depending on PA measurement Intervention vs. control Intervention group more likely to meet MVPA guidelines than the control group
Marigliano (2016) [57]	Quasi-experimental (Not reported)	General (70)	New York	Office of Management and Budget definition	In-person	None	Steps	Steps increased after 10 weeks
Melton (2016) [58]	Quasi-experimental (Not reported)	Prenatal patients or patients of reproductive age (328)	Southeast	Not defined	In-person (OB/GYN clinic)	None	% participants meeting guidelines	Intervention vs. control More people in the intervention group met MPA, VPA, ST, MVPA, and MVPA + ST guidelines than the control group at 6 months
Parker (2010) [59]	Pre-post (Not reported)	African American, overweight or obese (35)	South Carolina	Not defined	In-person (Church)	LIFE Project^e^	PA	PA increased after 10 weeks
Pullen (2008) [60]	RCT(Not reported)	Overweight and obese (21)	Midwest	Population < 2,500	Remote	Wellness for Women: Logging on to Lose Weight	Energy expenditure	Intervention group Energy expenditure decreased after 12 weeks Control group Energy expenditure increased after 12 weeks
Puma (2018) [61]	Pre-post (Health Belief Model, Transtheoretical Model)	Postpartum women (292)	Colorado	Frontier county	Hybrid (WIC centers)	Heart Smart Moms	PA	PA increased after 2 years
Sanchez (2021) [62]	Pre-post (Not reported)	Hispanic (50)	Washington	Not defined	In-person (Community spaces)	Eat Healthy, Be Active Communities	LPA, MPA, VPA	All PA measures increased after 6 weeks
Scarinci (2014) [63]	RCT (Social Cognitive Theory, Transtheoretical Model)	African American (565)	Alabama	Not defined	Hybrid (Community spaces)	None	Reported PA 5 or more days/week	Intervention PA increased at 12 months but was not maintained at 24 months Control PA increased at 12 and 24 months compared to baseline
Sherman (2007) [64]	Pre-post (Not reported)	Patients at Northeast Missouri Health Council Primary Care Clinic (75)	Missouri	Population < 17,000	Hybrid (Primary care clinic)	None	Steps	Step count increased after 6 months
Smith (2021) [27]	Pre-post (Not reported)	Cancer survivors (16)	Not reported	RUCC	In-person (Cancer care organization)	Better Exercise Adherence after Treatment for Cancer (BEAT Cancer)	MPA, VPA, MVPA	PA increased after 3 months
Wang (2020) [65]	Pre-post (Not reported)	General (177)	Alabama	Not defined	Hybrid	None	Steps	Steps increased after 4 days
West (2010) [66]	RCT (Not reported)	Medicare beneficiaries with diabetes living in disadvantaged areas (610)	New York	Not defined	Remote	Informatics for Diabetes Education and Telemedicine project (IDEATel)	Steps, “exercised regularly”	61% of intervention participants reported exercising regularly, 40% reported increasing their steps
Wyatt (2008) [67]	Pre-post (Cognitive Behavioral Theory)	Overweight (199)	New York	Not defined	In-person (Community spaces)	Calcium Weighs-In	Steps	Steps increased after 16 weeks
**Multi-level (k = 17)**								
Campbell (2002) [68]	RCT (Social Cognitive Theory, Transtheoretical Model)	Blue-collar workers (859)	North Carolina	Not defined	In-person (Worksite)	Health Works for Women	% participants reporting exercising, frequency of specific exercises per week, total MET min/week	Intervention Increased % of participants who reported exercising, increased times per week in jogging, cycling, ‘other’ exercise, and total MET min/week Control Increased times per week in aerobic activities, walking, jogging, aerobic dance, ‘other’ dance, aerobics classes, strengthening, flexibility Intervention vs. control Intervention group Increased strength & flexibility exercises/week at 6 and 18 months compared to the control group
Campbell (2012) [69]	Quasi-experimental (Social Cognitive Theory, Goal Systems Theory, Hope theory)	Overweight, low-income, minority groups (721)	North Carolina	Not defined	In-person (Community spaces)	HOPE Works (Health Opportunities, Partnerships, & Empowerment)	MVPA	Intervention PA increased after 12 weeks Control PA decreased after 12 weeks Intervention vs. control Intervention group increased PA compared to control
Fahs (2013) [70]	RCT (Transtheoretical Model, Socio-ecological Model, Moos Model)	General (117)	New York and Virginia	RUCC	In-person (Not reported)	Promoting Heart Health	Total Time Summary Index (TTSI), Energy Expenditure Summary Index (EESI), and Activity Dimension Summary Index (ADSI) scores	No change in PA in either group after 14 months No difference in PA change between groups
Folta (2019) [71]	RCT (Not reported)	Sedentary, overweight, midlife (194)	Montana and Nebraska	RUCA	In-person (Community spaces)	Strong Hearts, Healthy Communities	Steps, LPA, MVPA, walking	Intervention All PA measures increased after 6 months Control All PA measures increased after 6 months Intervention vs. control Intervention group increased walking more than the control group
Hopper (2017) [72]	Quasi-experimental (Not reported)	Minority group (236)	North Carolina	Not defined	In-person (Not reported)	Seeds of HOPE (Health, Opportunities, Partnerships, & Empowerment)	MPA, VPA, walking	PA increased in both groups after 6 months
Khare (2014) [73]	Pre-post (Not reported)	General (266)	Illinois	RUCA	In-person (Community spaces)	Heart Smart for Women (HSFW)	MPA, VPA, % participants meeting guidelines	MPA and VPA increased after 6 months and remained higher than baseline after an additional 6 months More people met guidelines after 6 and 12 months
Mier (2011) [74]	Pre-post (Stages of Change Model)	Spanish-speaking Mexican–American (18)	Texas	Not defined	In-person (Designated participant homes)	Vamos a Caminar	Walking	Steps increased after 3 months
O’Brien (2016) [75]	RCT (Social Cognitive Theory)	Older (24)	North Carolina	Not defined	Hybrid (Senior center and home)	Lose It	PA	Intervention PA increased after 12 weeks
Peterson (2005) [76]	RCT (Not reported)	Sedentary, midlife (44)	Midwest	Population < 25,000	In-person (Church)	Heart and Soul Physical Activity Program (HSPAP)	PA	Both groups increased PA after 3 months
Peterson (2013) [77]	Pre-post (Social Cognitive Theory, Transtheoretical Model)	Women seeking guidance for weight management at a primary care clinic (27)	NR	Frontier county	In-person (P{rimary care clinic)	Stay Alive with Five	MPA & VPA	All PA decreased at 6 and 12 months
Seguin-Fowler (2019) [78]	Pre-post (Not reported)	Overweight or obese, sedentary, midlife or older, Spanish-speaking Latinas (15)	Washington	RUCA	In-person (Community spaces)	Mujeres Fuertes y Corazones Saludables	MET min	PA decreased after 12 weeks
Tinius (2020) [79]	RCT (Not reported)	Pregnant (70)	Kentucky	Not defined	Hybrid (Convenient location chosen by the participant)	None	Steps, MET hours of LPA, MPA, VPA, household & caregiving, occupational, transportation, sport & exercise	All PA measures decreased after 17–28 weeks
Thomson^f^ (2016) [80]	RCT (Social Cognitive Theory, Transtheoretical Model)	Pregnant, African American (82)	Mississippi	Not defined	In-person (Home)	Delta Healthy Sprouts	PA	Intervention and control PA decreased after 5 months in both groups Intervention vs. control No difference in PA change between groups
Thomson^f^ (2018) [81]	RCT (Social Cognitive Theory, Transtheoretical Model)	African American mothers (82)	Mississippi	Not defined	In-person (Home)	Delta Healthy Sprouts	PA	Intervention Increased PA from 1 to 6 months postpartum but decreased PA at 12 months postpartum compared to 6 months postpartum Control Increased PA from 1 to 6 months postpartum and from 6 to 12 months postpartum Intervention vs. control No difference in PA change between groups
Walker^g^ (2009) [82]	RCT (Health Promotion Model)	Midlife (225)	Midwest	Not defined	Remote	Wellness for Women	MVPA, energy expenditure, strengthening, stretching, % meeting physical activity guidelines, % reaching 210 min MVPA/week	Intervention Increased all PA measures after 6 and 12 months compared to baseline Control Increased all PA measures after 6 and 12 months compared to baseline except energy expenditure decreased at 12 months compared to baseline Intervention vs. control More intervention participants reached 210 min MVPA/week after 12 months
Walker^g^ (2010) [83]	RCT (Health Promotion Model)	Midlife (225)	Midwest	Not defined	Remote	Wellness for Women	MVPA, strengthening, stretching	Intervention All PA measures decreased 6 and 12 months post-intervention compared to immediate post-intervention Control All PA measures decreased 6 and 12 months post-intervention except MVPA increased 6 months post-intervention compared to immediate post-intervention
Warren (2010) [84]	Quasi-experimental (Not reported)	Working women (188)	New York	Not defined	Hybrid (Worksite)	Small Steps Are Easier Together (SmStep)	Steps	Steps increased after 10 weeks

PA: Physical activity

MVPA: moderate-to-vigorous intensity physical activity

LPA: light-intensity physical activity

RCT: Randomized controlled trial

RUCA: Rural–Urban Commuting Area; 1–3 urban, 4–10 urban

RUCC: Rural–Urban Continuum Code

Office of Management and Budget definition: A Metro area contains a core urban area of 50,000 or more population, and a Micro area contains an urban core of at least 10,000 (but less than 50,000) population. All counties that are not part of a Metropolitan Statistical Area (MSA) are considered rural. [44]

^a^Reported results are only what was reported in the study. Not all studies reported differences between intervention vs. control groups, etc.

^b^Steps refers to the number of steps while walking is based on time

^c^Hagemen 2017 and Hageman 2022 are different analyses from the same intervention; 2017 compared groups starting immediately postintervention, 2022 analyzed a subgroup across the intervention and control groups at a long-term follow up

^d^General” refers to a population that has no strict eligibility criteria

^e^LIFE: L = Love, for self, family and God; I = Inspiration, from friends, God and family; F = Feedback; and E = Education, about dietary practices, daily physical activities, and discussions with health care providers

^f^ Thomson 2016 and Thomson 2018 are different analyses from the same sample of women; the intervention spanned from pregnancy through postpartum; 2016 analyzed participants while pregnant; 2018 analyzed participants postpartum

^g^ Walker 2009 and Walker 2012 are different analyses from the same intervention; 2009 was immediate postintervention and 2010 was a long-term follow-up

### Quality

Four studies were considered high quality (across three interventions), four were moderate quality, 14 were low quality, and 19 were very low quality. Based on the study design, 19 studies were initially rated/”graded” as high quality due to conducting randomized controlled trials, six as moderate quality due to quasi-experimental study designs, and 17 as low quality since they did not have a control group. The most common reasons for study quality being downgraded were that > 20% of the participants were lost to follow-up (k = 20 studies), and the intention-to-treat principle was not followed for statistical analyses (k = 21 studies). Study quality details can be found in Additional File 2.

### Feasibility, acceptability, strengths, and barriers

There was limited information available across studies regarding reported measurements of feasibility and acceptability as well as intervention strengths and barriers. Attendance sessions/protocol compliance was reported in ten studies at the individual level (64–92%) [27, 45, 46, 49, 55–57, 69, 79, 85]. Four studies reported on desirable intervention components (40% [daily text messages reporting their steps]—100% [educational brochures]) [27, 55, 69, 79]. For strengths, two studies reported their intervention was cost-effective. [51, 64] Another reported strength was using pre-existing relationships to recruit for and implement the intervention. [64] Participant-reported strengths were enjoying making new friends, feeling supported, and being aware of their lack of physical activity. [46, 55, 59, 69, 76, 78] Regarding barriers, six studies reported cost-related barriers, [27, 45, 60, 61, 67, 73] four reported staffing/interventionist issues, [27, 60, 61, 77] three reported insufficient time to reach desired intervention effects, [61, 68, 75] and two interventions reported problems with intervention logistics such as differences in community center spaces. [49, 61] Other barriers included family members not supporting the intervention, interventionist certification standards, and technical problems. [27, 75, 78].

### Individual vs. multi-level intervention findings

#### Individual

Twenty-four studies on 23 interventions were implemented at the individual-level (nine in-person, eight remote, and six hybrid). Ten interventions included virtual components such as web platforms, phone calls, text messages, or newsletters; eight were community-based and took place in public buildings such as churches, libraries, or recreation centers; three were based in a healthcare setting, and one was based in a Women, Infant, Children (WIC) Center. Generally, interventions provided education on physical activity guidelines, the benefits of physical activity, suggestions on how to be physically active, and proper techniques for specific exercises; guided goal-setting, or aimed to increase self-efficacy toward physical activity.

Nineteen interventions increased physical activity [27, 45–49, 51, 52, 56–59, 61–64, 66, 67, 86], and one intervention observed a subsequent decrease in physical activity 12 months post-intervention [48]. One intervention reported no change [53] but a subset of women with arthritis and obesity were found to increase their physical activity immediately post-intervention and at subsequent follow-ups when compared to baseline [54]. Two interventions reported mixed findings across physical activity outcomes: one reported an increase in meeting MVPA guidelines but a decrease in pedometer-measured number of steps [55], and the other reported increased minutes of MVPA but a decreased percentage of participants meeting MVPA guidelines. [50] The final intervention reported decreased physical activity [60]. All interventions that were delivered in a community setting increased physical activity. The intervention that observed no change in physical activity and the intervention that decreased physical activity were both delivered remotely [58, 73].

One successful, high-quality intervention was aimed toward prehypertensive rural women in Nebraska called *Wellness for Women: DASHing toward Health.* [52] The intervention was a randomized controlled trial delivered in a hybrid method in over 200 women. Participants received two 2-h in-person training sessions, one educational and another focused on goal-setting and using the Wellness for Women: DASHing toward Health web platform to track physical activity and diet. For 12 months, intervention participants received tailored newsletters (via web or print), individual goal-setting meetings, an exercise video, resistance bands, and encouraging text messages. All groups increased their physical activity from baseline to 6, 12, 18, and 24 months.

#### Multi-level

This review included 17 studies on 15 interventions incorporating multiple levels (11 in-person, three hybrid, one remote). One study provided follow-up data on an intervention [83], and another intervention reported results across two studies because they intervened on women from pregnancy through postpartum [80, 81]. Eight interventions were community-based and hosted in public spaces, two were implemented at worksites, two were home-based, two incorporated virtual components such as web platforms and newsletters, two were implemented at senior centers, and one was implemented in a healthcare setting. The majority of interventions aimed to change individual-level components, such as self-efficacy, with the interpersonal level by promoting strategies to enhance social support from peers, family members, and employers. Other strategies included interventions that combined individual-level components such as waiving fees for gym memberships, [79] encouraging participants to improve community-level factors via civic engagement [69], or making walking routes in the workplace [68].

Eleven interventions increased physical activity [68, 69, 72–77, 82, 84, 87], one reported no change, [70] and four decreased physical activity. [78–81] Notably, seven of the interventions that increased physical activity were community-based [49, 69, 72–74, 76, 82]. Also, of the interventions that observed decreases in physical activity, all prioritized a special population (2 prenatal, 1 postpartum, 1 overweight/obese). Of these interventions, two were delivered in-person [54, 79] and one was hybrid [49].

One successful, high-quality intervention, *Wellness for Women*, was a randomized controlled trial to increase physical activity and improve dietary habits in midlife women in the Midwest. [82, 83] The intervention lasted for 12 months and included over 200 participants. Intervention participants received 18 tailored newspapers to improve physical activity and diet based on individual survey responses at baseline (with specific newsletters dedicated to obtaining social support), an instructional exercise video, and a pedometer with encouragement from study staff to reach 10,000 steps/day across the intervention period. Participants also had a ‘commitment to a plan of action’ meeting every 3 months where they would choose 1–2 physical activity goals and 1 diet goal to achieve in a 3-month period. The control group received 18 generic newspapers, the exercise video, and the pedometer. Both groups increased their physical activity (energy expenditure and time spent in MVPA, strengthening, and stretching) at 6 and 12 months, except the control group decreased their energy expenditure at 12 months. At 6 and 12 months, more intervention participants met MVPA guidelines than the control group. Except for MVPA in the control group at 6 months post-intervention, both groups decreased all physical activity outcomes at 6 and 12 months compared to immediately post-intervention.

### Quantitative analysis

**Fig. 2:**
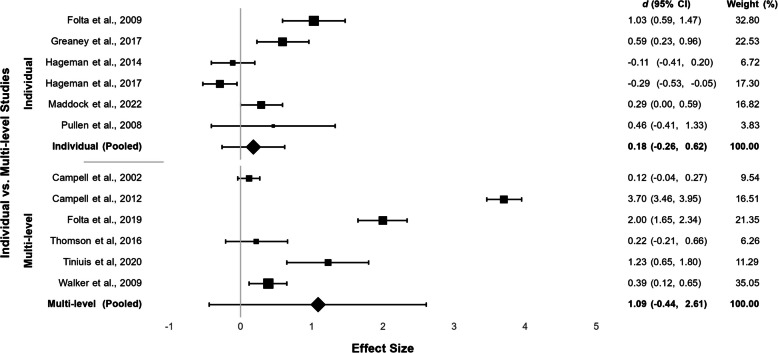
Individual vs Multi-level studies

Six individual and six multi-level studies were included in the quantitative analysis. Multi-level interventions (*d* = 1.09 [−0.44, 2.61], I^2^ = 99.26%) had a larger pooled effect than individual interventions (*d* = 0.18 [−0.26, 0.62], I^2^ = 86.74%). See Fig. 2.

## Discussion

We found that physical activity interventions in rural US women targeted either individual (61%) or multi-level (39%) changes and, generally, most increased physical activity. Intervention delivery (i.e., in-person, remote, or hybrid) or format (e.g., community-based, worksite) did not appear to make a difference to intervention success. Since 80% of total interventions were successful, it is difficult to disentangle which intervention components were critical to increase physical activity. However, the high success rate suggests that rural women in the US may be receptive to interventions that increase their physical activity, making future research on effectiveness-implementation hybrid trials a reasonable next step. This is exemplified by one study which reported that they met their recruitment goal of 75 subjects within two days [55]. Only five interventions (13%) observed a decrease in physical activity, most of which were in pregnant and postpartum women who were undergoing a critical life transition where physical activity typically declines. [80, 88] Studies included a wide range of states and special populations, including pregnant and postpartum women, the elderly, underrepresented racial groups, those with low socioeconomic status, blue-collar workers, and populations with specific medical considerations. According to our quantitative analysis, and in line with previous research in general populations, multi-level interventions resulted in greater increases in physical activity [89, 90]. However, these results must be interpreted cautiously as only twelve studies were included in this analysis, of which eight were low or very low quality according to the GRADE approach. Additionally, as expected, high I^2^ values suggest that the populations used in the meta-analysis differ.

Most studies did not report concrete data on strengths and barriers for their interventions, however, we identified a few important trends. Participants typically valued feeling supported, whether that be by research staff, fellow participants, or individuals in their personal social circles. Next, to ensure interventions are appropriately implemented, researchers should carefully consider the responsibilities, needs, and training of the interventionists. For example, one study discussed that not all participants received their counseling sessions as planned because the interventionist, a nurse educator, had left their position [77]. Another study used WIC educators to implement the intervention, but they reported several interventionist issues, such as low self-efficacy to counsel study participants, high staff turnover, limited visit time, and unbalanced workloads [61]. Additionally, several studies reported that their intervention was not cost-effective [27, 45, 60, 61, 67, 73]. These characteristics are important to consider to make not just effective, but scalable and sustainable interventions. Additionally, future studies should include process and implementation evaluations, feasibility and acceptability measures, community-engagement efforts, and a clear, transparent report of intervention development steps in their protocols to better inform and refine interventions.

This review identified several research gaps. First, we need more high-quality interventions designed to test the efficacy of multi-level interventions while still benefitting all US, rural women participants. This includes randomized controlled trials, quasi-experimental, crossover, and delayed intervention designs focused on promoting physical activity in rural US women. Most interventions increased physical activity; however, many were of low or very low quality, and several used a pre-post study design. Thus, it is difficult to make firm conclusions with the current evidence. Next, although several states and regions were represented, many other areas, such as the Southwest and Appalachia, had little or no representation. This is important because each geographic area of the country will likely need special considerations depending on climate, resources, and cultural context. Next, a notable gap in the literature is that more than half of the included studies did not define rurality. The rurality definition is important to properly reproduce interventions across populations. For example, strategies used in an intervention that was successful in a population that defined rurality as < 25,000 may not be as effective or feasible in a frontier county due to spatial, resource, and intervention delivery capacity considerations. Additionally, the interventions in this review that used technology are generally outdated, given current advances in technology and digital literacy. Specifically, nine of the included interventions were implemented either before or within 2008, the first year popular smartphones were released. This is important because the adoption of smartphones has drastically increased, and the use of electronic health applications has emerged prominently in recent years [91, 92]. Further, all of the interventions were implemented before the COVID-19 pandemic. This has important implications as digital literacy has typically increased, including in rural populations, since the pandemic, and digital interventions may be particularly useful for intervening in hard-to-reach populations [93]. Additionally, during the pandemic, the use and acceptability of remotely-delivered electronic health services improved in rural populations [94]. Therefore, considering updated technological intervention delivery methods may be useful in future intervention designs. Finally, all multi-level interventions in this review targeted changes across socio-ecological levels at the same time, primarily by increasing self-efficacy toward physical activity while promoting strategies to improve social support. Recommendations to advance and improve multi-level interventions include introducing a higher-level change targeting upstream determinants, such as building safe walking paths and bike lanes, followed by lower-level changes, such as raising community awareness or motivation to use the new resources [95]. Although complicated and expensive, these types of multi-level interventions may be helpful to increase and maintain physical activity changes in rural populations. However, there are no available studies testing such strategies.

Although this review identified important characteristics of physical activity interventions in rural US women, it is not without limitations. First, most studies were of low and very low quality. We could not make definitive conclusions on effect sizes due to the small number of studies that were randomized controlled trials and the high number of low-quality studies. Also, there were no interventions that were implemented specifically at the interpersonal, community, organizational, or policy level that met our eligibility criteria, so we cannot provide any insight into physical activity interventions that were only implemented at one of these levels. Finally, we could not make any conclusive statements about the feasibility and acceptability of interventions across different socio-ecological levels since these measurements were minimally reported.

## Conclusion

We summarized physical activity interventions in rural women in the US from the socio-ecological perspective. Our findings suggest that both individual and multi-level interventions are effective at increasing physical activity in this population. Meta-analysis results show that multi-level interventions may yield greater increases in physical activity; however, caution must be exercised with these results, as the confidence intervals of the pooled effects of both approaches overlap. Clearer definitions of rurality and more high-quality interventions, including randomized controlled trials, would strengthen this conclusion. Additional important considerations for designing interventions include ensuring participants feel supported; utilizing interventionists who have the time, stability, and confidence to appropriately conduct the intervention; and assessing and achieving cost-effectiveness. Future directions that should be prioritized for research include gaining more representation from across the country and integrating updated technology and platforms into interventions. Further, studies reporting on their interventions should include feasibility, acceptability, strengths, and barriers in their manuscripts.

## Supplementary Information

Supplementary Material 1.

Supplementary Material 2.

## Data Availability

The datasets generated and analyzed during the current study are available in the Open Science Framework repository, https://osf.io/64q78/files/osfstorage.
