# Supplementary material for: A scoping review comparing individual and multi-level physical activity interventions in rural women in the United States
Source: BMC Public Health. 2025 Dec 3;25:4211. doi: 10.1186/s12889-025-24018-y (PMC12676838; doi:10.1186/s12889-025-24018-y)
Supplement: Supplementary file 1 — Supplementary Material 1. [file 12889_2025_24018_MOESM1_ESM.pdf]

Additional file 1. PubMed Search Strategy.

("physical activity"[Text Word]) OR (exercise[MeSH Terms])) OR (exercise*[Text Word])) OR ("physical exercise*"[Text Word])) OR (exercise therapy[MeSH Terms])) OR (sports[MeSH Terms])) OR (exercise therapies[MeSH Terms])) OR (sports[Text Word])) OR (physical exertion[MeSH Terms])) OR ("physical exertion*"[Text Word])) OR (exercise movement techniques[MeSH Terms])) OR ("exercise movement technique*"[Text Word]) OR (resistance training[MeSH Terms])) OR ("resistance training"[Text Word])) OR (circuit-based exercise[MeSH Terms])) OR ("circuit-based exercise"[Text Word])) OR (aquatic therapy[MeSH Terms])) OR ("aquatic therapy"[Text Word])) AND (((intervention*[Text Word]) OR (treatment outcome[MeSH Terms])) OR ("treatment outcome*"[Text Word])) AND ((((((((((((rural health[MeSH Terms]) OR (rural[Text Word])) OR ("rural health"[Text Word])) OR (rural population[MeSH Terms])) OR ("rural population*"[Text Word])) OR ("rural area*"[Text Word])) OR ("poverty area*"[Text Word])) OR (rural health service[MeSH Terms])) OR ("rural health service*"[Text Word])) OR ("rural health center*"[Text Word])) OR (hospitals, rural[MeSH Terms])) OR ("rural hospital*"[Text Word])) OR ("rural environment*"[Text Word])) AND ((((women[MeSH Terms]) OR (women[Text Word])) OR (woman[Text Word])) OR (female*[Text Word]))
