# Supplementary material for: A scoping review comparing individual and multi-level physical activity interventions in rural women in the United States
Source: BMC Public Health. 2025 Dec 3;25:4211. doi: 10.1186/s12889-025-24018-y (PMC12676838; doi:10.1186/s12889-025-24018-y)
Supplement: Supplementary file 2 — Supplementary Material 2. [file 12889_2025_24018_MOESM2_ESM.pdf]

Additional File 2. Study Quality.

Author	Year	Study design	Blinding	Loss to follow-up	Intention-to-treat principle	Outcome Reporting	Physical Activity Measurement	Recruitment bias	Sample size	Fidelity/Adherence	Quality
Befort	2010	low	maintain	downgrade	maintain	maintain	maintain	maintain	maintain	maintain	very low
Befort	2012	low	maintain	maintain	downgrade	maintain	maintain	maintain	maintain	maintain	very low
Campbell	2002	high	maintain	downgrade	downgrade	maintain	downgrade	maintain	maintain	maintain	very low
Campbell	2012	moderate	maintain	downgrade	maintain	maintain	maintain	downgrade	maintain	maintain	very low
Edwards	2021	low	maintain	upgrade	downgrade	maintain	maintain	maintain	maintain	maintain	low
Fahs	2013	high	maintain	downgrade	downgrade	downgrade	maintain	maintain	maintain	maintain	very low
Fazzino	2017	low	maintain	maintain	downgrade	maintain	maintain	maintain	maintain	maintain	very low
Folta	2009	high	downgrade	maintain	maintain	maintain	maintain	maintain	maintain	maintain	moderate
Folta	2019	high	maintain	downgrade	maintain	maintain	maintain	maintain	maintain	maintain	moderate
Greaney	2017	high	maintain	downgrade	downgrade	maintain	maintain	downgrade	maintain	maintain	very low
Griffin	2018	low	maintain	downgrade	downgrade	maintain	downgrade	maintain	maintain	maintain	very low
Hageman	2014	high	maintain	maintain	maintain	maintain	maintain	maintain	maintain	maintain	high
Hageman	2017	high	maintain	downgrade	maintain	maintain	maintain	maintain	maintain	downgrade	low
Hageman	2022	high	maintain	downgrade	downgrade	maintain	maintain	maintain	maintain	maintain	low
Hopper	2017	moderate	maintain	downgrade	downgrade	maintain	maintain	maintain	maintain	downgrade	very low
Khare	2014	low	maintain	downgrade	downgrade	maintain	maintain	maintain	maintain	maintain	very low
Khare	2021	low	maintain	maintain	downgrade	maintain	maintain	maintain	maintain	maintain	very low
Maddock	2022	high	maintain	downgrade	maintain	maintain	maintain	maintain	maintain	downgrade	low
Marigliano	2016	moderate	maintain	maintain	downgrade	maintain	maintain	maintain	maintain	maintain	low
Melton	2016	moderate	maintain	maintain	maintain	maintain	downgrade	maintain	maintain	maintain	low
Mier	2011	low	maintain	maintain	maintain	maintain	maintain	maintain	maintain	maintain	low
O'Brien	2016	high	maintain	maintain	downgrade	maintain	downgrade	maintain	maintain	maintain	low
Parker	2010	moderate	maintain	maintain	downgrade	downgrade	maintain	maintain	maintain	maintain	very low
Peterson	2005	high	maintain	upgrade	maintain	maintain	maintain	downgrade	maintain	maintain	high
Peterson	2013	low	maintain	upgrade	downgrade	maintain	maintain	maintain	maintain	maintain	low
Pullen	2008	moderate	maintain	downgrade	maintain	downgrade	maintain	maintain	maintain	downgrade	very low
Puma	2018	low	maintain	downgrade	downgrade	maintain	downgrade	maintain	maintain	maintain	very low
Sanchez	2021	low	maintain	upgrade	maintain	maintain	downgrade	maintain	maintain	maintain	low
Scarinci	2014	high	maintain	downgrade	downgrade	maintain	maintain	maintain	maintain	maintain	low
Seguin-Fowler	2019	low	maintain	downgrade	maintain	maintain	maintain	maintain	maintain	downgrade	very low
Sherman	2007	low	maintain	maintain	maintain	maintain	maintain	maintain	maintain	maintain	low
Smith	2021	low	maintain	maintain	maintain	maintain	maintain	maintain	maintain	maintain	low
Thomson	2016	high	downgrade	downgrade	downgrade	maintain	maintain	maintain	downgrade	maintain	very low
Thomson	2018	high	downgrade	maintain	downgrade	maintain	maintain	downgrade	downgrade	maintain	very low
Tinius	2020	high	downgrade	maintain	downgrade	maintain	maintain	maintain	maintain	maintain	low
Walker	2009	high	maintain	upgrade	maintain	maintain	maintain	downgrade	maintain	maintain	high
Walker	2010	high	maintain	upgrade	maintain	maintain	maintain	downgrade	maintain	maintain	high
Wang	2020	low	maintain	upgrade	maintain	maintain	maintain	maintain	maintain	maintain	moderate
Warren	2010	low	maintain	downgrade	maintain	maintain	maintain	maintain	maintain	downgrade	very low
West	2010	high	maintain	maintain	maintain	maintain	downgrade	maintain	maintain	maintain	moderate
Wyatt	2008	low	maintain	downgrade	downgrade	maintain	maintain	maintain	maintain	maintain	very low

Study design: high if randomized controlled trial, moderate: non-randomized/quasi randomized controlled trial, low: no control groups in trial
Blinding: maintain: those recording outcomes, and/or data analysts are blinded to which arm participants are allocated; or unclear; downgrade: those recording outcomes, and/or data analysts are aware of which arm participants are allocated
Loss to follow-up: upgrade: if at least 90% of participants followed up; maintain: if at least 80% of participants followed up; downgrade: if 20% of more of participants were lost to follow-up
Intention-to-treat principle followed: maintain: if intention-to-treat principle was followed or unclear; downgrade: if the intention-to-treat principle was not followed in a RCT
Outcome reporting: maintain: all described measurements were fully reported; downgrade: not all physical activity measurements or other defined measurements (e.g., if objectives mentioned feasibility would be measured) were reported
Physical activity measurement: maintain: objective or validated measures were used or unclear; downgrade: measures were not validated
Recruitment bias: maintain: no selection bias evident (i.e., groups are comparable at baseline); downgrade: selection bias present (i.e., groups are different at baseline)
Sample size: maintain: power calculation reported or unclear; downgrade: recruitment goals not met
Fidelity/adherence: maintain: fidelity and adherence was measured or unclear; downgrade: fidelity and adherence are below 80%
